# Sex differences in the cognitive performance in adults: role of impaired sleep

**DOI:** 10.5935/1984-0063.20210022

**Published:** 2022

**Authors:** Luciane de Souza Medeiros, Flavia H. Santos, Alana Peixoto Almeida, Davyd M.O. Alves, Renan Remaeh Rocca, Sergio Tufik, Adriana Ximenes-da-Silva

**Affiliations:** 1 Universidade Federal de Alagoas, Instituto de Ciências Biológicas e da Saúde - Maceió - AL - Brazil.; 2 University College Dublin, UCD School of Psychology, D04 V1W8 - Dublin - Ireland.; 3 Universidade Integrada Tiradentes, Psicologia - Maceió - AL- Brazil.; 4 Universidade Federal De São Paulo, Psicobiologia - São Paulo - SP - Brazil.

**Keywords:** Sex Differences, Working Memory, Attention, Sleep

## Abstract

**Objectives:**

Poor sleep quality negatively affects cognitive performance. However, there are limited data on sex differences in functional outcomes of impaired sleep on cognition. Therefore, the aim of this study was to evaluate the association between sleep quality and performance of men and women in cognitive tests.

**Material and Methods:**

After screening, 97 individuals with and without insomnia complaints participated of this study. Behavioral scales were evaluated using a number of instruments and the sleep pattern was recorded by actigraph. Subsequently, the participants were submitted to visuospatial/verbal working memory (WM), visual attention, and psychomotor vigilance tests (PVT).

**Results:**

The actigraphic recordings indicated that men sleep later (d=-0.56, p<0.05), fall asleep faster (d=0.42, p<0.05), showed shorter sleep duration (d=0.53, p<0.05), and more sleep fragmentation than women (d=-0.41, p<0.05). The performance in the cognitive tasks also showed sex differences: the men showed better performance in the visuospatial short-term memory (d=-0.78, p<0.05); verbal (d=-0.61, p<0.05), and visuospatial-WM tests (d=-0.84, p<0.05); they also responded faster in the PVT (d=0.69, p<0.05), although made more mistakes (d=-0.85, p<0.01). Longer sleep latency was associated with poor performance in visual attention (r=0.52, p<0.05) and verbal memory tasks (r=-0.30, p<0.05) in men.

**Conclusion:**

Our results suggest that difficulty in falling asleep was associated with cognitive impairment, especially in men. Sex differences in sleep quality and cognitive skills should be taken into account in future research in this field.

## INTRODUCTION

The sleep amount in adults varies significantly among individuals, probably because of genetic differences, although, other factors, like age and sex might underscore this variation. Physiological changes in sleep patterns and a decrease in sleep time are expected with aging, however, the effects of sex are less known. Some studies have indicated that women have greater sleep latency (SL)^1^ and total sleep time (TST) than men^2^, especially between the age of 25 and 64 years^3^. However, independent of sex, TST short are becoming frequently in the modern civilization, especially in the full-time workers^4^.

Sleep is essential for physical and mental health, and productivity. However, due to the constant pressure from work, social, and family activities, a large proportion of the adult population has a total of 6 sleeping h/d or even less^4^. According to the National Sleep Foundation (NSF) young adults need around 7 to 9 hours of sleep per day^5^. A small part of the population follow the short (<5h) and long sleeper (>10h) patterns found in adults^6^. Sleep loss leads to cognitive and behavioral performance dysfunction and deteriorates in a dose-dependent manner^7^. There are interindividual differences in the basal sleep need and the vulnerability to the cognitive effects of sleep loss^8^. Although it is not clear if men and women respond cognitive differently to poor sleep quality, sex and gender differences may underlie the differential risk for sleep disorders. Chronic difficulties initiating or maintaining sleep (insomnia) are more frequently found in women, and are associated with anxiety and/or depressive symptoms^9^.

Lapses of attention and a decline in different cognitive domains have been observed in healthy volunteers submitted to a sleep deprivation (SD)^10^ and in patients with chronic insomnia^11^. However, conflicting results have been observed, especially in relation to working memory (WM) tests^10-12^. Since WM involves multicomponent cognitive processes, this outcome depends on the specific component of the system assessed. Moreover, discrepant results may be due to the fact that some studies did not consider age-related individual variability and the role of sex in TST^13,14^.

Although there is evidence for sex differences in verbal and spatial memory, with women performing better in verbal tasks and men in visuospatial tasks^15,16^, some underlying aspects need to be considered. For instance, the magnitude of sex-related differences in the performance of verbal and spatial tasks depends on age, the tests used and the type of ability that is measured. In several studies, the effect sizes are small (Cohen d<0,02) or did not have significant differences^17,18^. Moreover, the sleep time duration is not one of the variables considered in studies that evaluate memory performance of men and women.

As there are sex differences in sleep time and in some cognitive abilities, it is possible presume that physiological responses to SD are different in the men and women. Some studies have suggested that women are more resilient than men to external stressors or SD^2,19^. Although, Rångtell et al. (2018)^20^ showed that men’s WM performance remained unaffected by sleep loss after a single night SD. In another study, one night of SD did not affected performance in different tests of executive functions of the group of young adults formed by men and women^21^. However, as yet only a few studies have examined the role of sex in vigilances tasks or other cognitive aspects following SD. In most articles, the results of tests performed by men and women are evaluated together. Furthermore, sometimes the numbers of either men or women are small, or there are other variables such as interindividual differences and age which may confuse or mask the interpretation of the results^14,21-24^. Therefore, in spite of many studies about the effects of the SD on cognition, there are limited data on sex differences in functional consequence of impaired sleep. Moreover, these studies are usually performed in strictly controlled laboratory settings, with different paradigms of wake-sleep cycle, and it is therefore necessary to develop studies in more naturalistic environments. In this study, we investigated sex differences in quality of sleep and in cognitive abilities, and we hypothesized that impaired sleep quality could affect the performance of men and women in attention and WM tests differently. Furthermore, considering that sleep complaints are often associated with depressive and/or anxiety symptoms, we evaluated these symptoms to eventually exclude confounding factors.

## MATERIAL AND METHODS

### Volunteers

Volunteers were recruited from fliers, official website of University and in local media. This study was approved by the Ethical Committee of the Universidade Federal de Alagoas (CAAE: 15357913.0.0000.5013). After pre-screening by telephone, 118 individuals (20-45 years old) signed informed consent for the study. Data of 21 were not used for the statistical analyzes because 5 did not complete the study, 7 became ill or took psychoactive medication during the week of the study, and 9 were excluded during the screening for drugs^25^ or cognitive decline evaluated by mini-cog test^26^. Thus, we will show the results for 50 women and 47 men non-smokers. All volunteers met the following criteria: had ≥11 years of schooling; did not have acute medical conditions or conclusive diagnoses of sleep/psychiatric disorders; did not have low visual/hearing acuity; except if corrected by wearing glasses/hearing aid; did not misuse alcohol or use medication that induces sleepiness.

### Procedures

The volunteers attended the laboratory on two occasions: (1^st^) to complete a series of self-report measures assessing sleep quality^27,28^, circadian rhythm^29^, and the use of psychoactive substances^25^. The body mass index of the volunteers was calculated based on height and weight measurements; (2^nd^) the participants performed the automated WM assessment (AWMA), the D2 test, and the psychomotor vigilance task (PVT), in this order. They also completed self-report forms regarding symptoms of depression and anxiety^30,31^ in order to control for possible confounding factors in the performance cognitive.

During the 1^st^ session (Monday or Tuesday), the volunteers were instructed on how to complete the sleep diaries and use the actigraph, which they had to wear for 8-10 days. They were told to maintain their normal daily routines and food habits, including the consumption of caffeinated beverages throughout the experiment. However, 24 hours before the second session they were asked to avoid ingestion of alcohol and any medication that affects alertness or sleep. The cognitive assessment took place on the Wednesday or Thursday of the following week, and volunteers could choose a time to attend (10h or 12h or 14h). They were instructed to choose the time of their preference, considering the period when they were most alert. Each evaluation lasted around 2 hours, and the consumption of stimulants beverages were prohibited for the 6 hours before the test.

### Actigraphy recording

The actigraph used was the Mini Motionlogger Actigraph - Basic 32C (Ambulatory Monitoring, Inc., Ardsley, U.S.). Data were collected using the “zero crossing mode” in one-minute epochs. The following sleep parameters were analyzed: TST in hours; SL and wake after sleep onset (WASO) in minutes; sleep efficiency (SE) as a percentage; sleep fragmentation (SF); and number of awakenings (AW). The wake-sleep cycle parameters were: sleep time (STime), end time (ETime), and midpoint of sleep time (MTime) mean and variability (standard deviation). Moreover, information about rhythm regularity (interdaily stability), synchronization (intradaily variability), and rhythm amplitude (relative amplitude) were collected.

### Questionnaires

Alcohol, smoking and substance involvement screening test (ASSIST): a questionnaire to detect substance use, screening for all levels of problem or risky substance use in adults^25^. The reliability of the instrument was good (Cronbach’s alpha of 0.80 to alcohol, 0.79 to cannabis, and 0.81 to cocaine). The cutoff adopted to select participants was >11 points for alcohol and >4 for other substances.

Pittsburgh sleep quality index (PSQI): a self-rated questionnaire that assesses sleep quality and disturbances over a 1-month period. A PSQI global score of >5 is indicative of poor sleep quality. The seven-component scores of the Brazilian Portuguese version^27^ had an overall reliability coefficient (Cronbach’s α) of 0.82.

Epworth sleepiness scale (EES): a self-report questionnaire with 8 questions that assess the level of daytime sleepiness. An ESS score >10 suggests excessive daytime sleepiness. The 8-item scores of the ESS in Brazilian Portuguese^28^ had an overall reliability coefficient of 0.83.

The Munich chronotype questionnaire (MCTQ): a self-report questionnaire to assess an individual’s phase of entrainment on work and work-free days. The MCTQ contains 29 questions about sleep and waking times and identifies the individual’s chronotype based on the midpoint between sleep onset and offset on work-free days (mid-sleep). The correlation between the Horne and Ostberg morningness-eveningness questionnaire (MEQ) was r=-0.73 to mid-sleep times^29^.

The Beck depression inventory - 2^nd^ edition (BDI-II): a 21-item, self-report measure of the severity of depressive symptoms^30^. The suggested thresholds for levels of severity were: 0-13, minimal/no depression; 14-19, mild depression; 20-28, moderate depression; and 29-63, severe depression. The intraclass correlation coefficient of the BDI-II was 0.89, and the Cronbach’s alpha coefficient of internal consistency was 0.93.

The Beck anxiety inventory (BAI): a 21-item, self-report measure of the severity of anxiety symptoms. A total score of 0-21 is considered very low anxiety, 22-35 is moderate, and 36 or higher is severe. The reliability^31^ for psychiatric samples was α=0.91, for clinical samples was α=0.86, and for non-clinical samples α=0.86.

### Cognitive tasks

The Automated Working Memory Assessment (AWMA): a computer-based standardized battery comprising 12 tests that evaluate verbal and spatial memory^32^. The Portuguese version used was adapted by Santos and Engel (2008)^33^. The test was applied individually to each volunteer in a quiet room by a trained technician. There are 4 subtests that evaluate verbal short-term memory (the results given as mean composite verbal short-term memory scores/V-STM), 4 visuospatial short-term memory (mean composite visuospatial short-term memory scores/VS-STM), 4 verbal WM (mean composite verbal WM scores/V-WM), and 4 visuospatial WM (mean composite visuospatial WM scores/VS-WM). The tests usually start with 3 items, with 1 item being added until the participant is unable to recall them.

The D2 test: a neuropsychological test that evaluates sustained and selective attention^34^. The individual has 20 seconds to scan each line and mark with a cross only the letters “d” with two dashes above or below it in any order. Are considered errors when the number of target symbols are not marked or when the number of non-target symbols (letter “p”) or “d” with one or three marks are marked. The measures used were: 1) total number of characters processed (TN), the number of characters processed before the end of each trial; 2) total correctly processed (TN-E), the total number of characters processed minus total errors made; 3) the percent of errors (E%), the number of errors divided by the number of characters processed.

The psychomotor vigilance task (PVT): used to test sustained vigilance and reaction time^35^. It measures the speed with which subjects respond to a random visual stimulus over a period of 10 minutes. The variables evaluated were: mean reaction time (RT), the time taken to interrupt the visual stimulus; false start (FS) is the response with a reaction time of less than 100ms and lapses are the response longer than 500ms.

### Statistical procedures

Data from the actigraphic recordings were averaged over the 8 days prior to the 2^nd^ session. The analyses of cognitive tests and behavioral scales were cross-sectional studies obtained with the use of XLSTAT software and StatSoft version 6, considering statistical significance to be p<0.05. To evaluate the sex effect, one-way multivariate analyses of variance (MANOVA) were performed, using Tukey post-hoc analysis when necessary. Cohen d effects sizes were calculated for sleep and cognitive parameters. The effects sizes were considered positive when W>M and negative when M>W. The missing data were due problems with the equipment or incorrect completion of the scales.

To test for associations between the variables, Spearman (ρ) or Pearson’s correlation coefficients (r) were calculated. The sex variable was dummy coded as 1 for women and 2 for men. If there was a significant correlation between the variables the ANCOVA would be performed. Also, the R^2^ (coefficient of determination) it was calculated. The R^2^ indicates the % of the variability of the dependent variable which is explained by the explanatory variables (quantitative and qualitative independent variables). The sex was always the qualitative variable, whereas age, BDI score, and actigraphic parameters (TST, SL, and SF) as the independent quantitative variables. To explore the relationships among actigraphic parameters and cognitive tests by sex, we conducted partial correlations covaried for age.

## RESULTS

### Demographic data

The age, education and body mass indices (BMI) data for each group are presented in Table 1. No statistical differences [Wilks’ Λ=0.98, F(3, 90)=0.56, p=0.65] were found by the one-way MANOVA between the sex for the variables of age (p=0.85), education (p=0.31), and BMI (p=0.51).

**Table 1:** Demographic information and clinical characteristics by sex.

	Men (n=47)			Women (n=50)		
	Age (y)	Education (y)	BMI (kg/m2)	Age (y)	Education (y)	BMI (kg/m2)
Min	20.0	12.0	17.4	20.0	12.0	15.7
Q1	23.0	15.0	23.0	23.0	15.0	20.3
Q2	28.0	16.0	25.8	27.0	16.0	24.2
Q3	38.0	17.0	27.9	36.0	17.0	27.9
Max	45.0	22.0	36.0	44.0	22.0	37.1
Mean	29.9	15.8	25.7	30.2	16.0	24.7
SD	8.0	2.3	3.9	7.7	1.9	5.1

Notes: First quartile (Q1), second quartile (Q2) or median, third quartile (Q3); Minimum (Min); Maximum (Max); Standard deviation (SD); Age and education in years (y); Body mass indices (BMI). p>0.05.

### Sleep data

We did not found a significant difference in the subjective evaluation of sleep quality, daytime sleepiness, and the chronotype (mid-sleep) between the sex [Wilks Λ=0.98, F(3, 93)=0.58, p=0.62]. On the other hand, there were statistical difference in the sleep-wake cycle [Wilks’ Λ=0.85, F(6, 89)=2.51, p<0.05] as sleep parameters recorded by actigraphy [Wilks’ Λ=0.84, F(6, 90)=2.77, p<0.05]. The post-hoc tests are listed in Table 2. The women reported significantly more depressive symptoms than men [Wilks’ Λ=0.92, F(2, 94)=3.61, p=0.03].

**Table 2:** Sleep quality, daytime sleepiness and mid-sleep by MCTQ; behavioral scales; sleep-wake cycle and sleep parameters by sex.

		Men (n=47)					Women (n=50)					
		**Min**	**Max**	**Mean**	**SD**		**Min**	**Max**	**Mean**	**SD**	**d**	**p**
**Subjective evaluation**	**PSQI**	0	13	6.2	2.7		3.0	12.0	6.7	2.2	0.20	0.3
	**Epworth**	2	21	9.6	3.9		2.0	22.0	10.2	4.6	0.14	0.5
	**MCTQ**	1.2	7.0	4.1	1.2		1.1	7.7	3.9	1.5	-0.15	0.6
**Behavioral scales**	**BAI**	0	30	8.3	6.6		2.0	24.0	8.6	5.8	0.05	0.8
	**BDI**	0	25	9.2	7.1		1.0	26.0	12.5	7.3	0.46	<0.05
**Sleep-wake cycle by actigraphy**	**STIME**	21.5	27.0	24.3	1.1		21.2	26.2	23.7	1.06	-0.56	<0.01
	**ETIME**	5.4	11.0	7.2	1.2		5.3	10.5	7.07	1.13	-0.11	0.5
	**MPOINT**	1.45	7.1	3.8	1.1		1.9	6.2	3.38	0.98	-0.40	0.06
	**IS**	0.10	0.82	0.54	0.18		0.33	0.81	0.60	0.11	0.41	<0.05
	**IV**	0.34	0.84	0.52	0.12		0.35	0.70	0.53	0.08	0.10	0.8
	**RA**	0.51	0.94	0.79	0.09		0.44	0.94	0.83	0.09	0.44	<0.05
**Sleep parameters** **by actigraphy**	**TST**	3.8	8.0	5.8	0.8		2.1	8.4	6.2	1.0	0.53	<0.05
	**SL**	5.3	57.4	16.3	10.9		3.2	61.9	21.7	14.3	0.42	<0.05
	**SE**	64.5	96.1	87.2	7.3		48.9	97.2	89.2	8.3	0.26	0.2
	**WASO**	15.8	160.6	51.7	32.6		9.0	129.2	44.1	29.5	-0.25	0.2
	**SF**	2.1	10.2	4.6	2.0		1.2	9.0	3.8	1.8	-0.41	<0.05
	**AW**	6.8	30.8	15.1.	5.8		21.2	26.2	13.3	5.6	-0.31	0.1

Notes: MANOVA, followed by a posteriori Tukey. Cohen’s d effects sizes were considered positive when W>M and negative when M>W. Minimum (Min); Maximum (Max); Standard deviation (SD). Pittsburgh Sleep Quality Index (PSQI), Munich Chronotype Questionnaire (MCTQ. Number of volunteers (N); Sleep Time (STime), End time (ETime), Midpoint of sleep time (MTime). Interdaily Stability (IS), Intradaily Variability (IV), Relative Amplitude (RA).Total Sleep Time (TST) in hours; Sleep Latency (SL) and Wake After Sleep Onset (WASO) in minutes; Sleep Efficiency (SE) in percentage; Sleep fragmentation (SF), Awakenings number (AW).

### Correlation analyses between the variables

Statistically significant correlation data between the variables are summarized in the Table 3 and the Table 4. Table 5 displays the significant partial relationship between actigraphic parameters and cognitive tests separated by sex while controlling for age. Only parameters statistically significant were shown in the Tables 4 and 5.

**Table 3:** Correlation coefficients between the demographic variables, BAI and BDI scores and sleep variables.

	Sex	Age	Education (Y)	BAI	BDI
**Actigraphic parameters:** **STIME** **ETIME** **MPOINT** **TST** **SL** **WASO** **SE-SD** **SF** **AW**	0.30 -- 0.22 -0.24 -- -- -- 0.21 --	-0.42 -0.30 -0.39 -- -- -- -- -- --	-- -- -- 0.21 -- -- -- -- --	-- -- -- -- -- 0.23 0.23 0.22 0.26	-- -- -- -- 0.26 -- -- -- --
**Subjective sleep evaluation:** **PSQI global score** **Epworth score** **MCTQ (mid-sleep)**	-- -- --	-- -0.21 -0.47	-- -0.33 -0.21	0.23 0.27 --	0.27 -- --

Notes: All correlation coefficients are statistically significant (p<0.05); Sleep time (STime); End time (ETime); Midpoint of sleep time (MTime); Total sleep time (TST); Sleep latency (SL); Wake after sleep onset (WASO); Sleep efficiency (SE); Sleep efficiency standard-deviation (SE-SD); Sleep fragmentation (SF); Number of awakenings (AW); Beck anxiety inventory (BAI); Beck depression inventory (BDI); Pittsburgh sleep quality index (PSQI); Munich chronotype questionnaire (MCTQ).

**Table 4:** Correlation coefficients between the demographic variables, BAI, and BDI scores, sleep variables and cognitive tests.

	AWMA (N=96)							PVT (N=86)		
	V- STM	VS-STM	V- WM	VS- WM	D2-TEST (N=94)			RT	FS	Lapses
					TN	TN-E	E%			
**Sex**	--	0.38	0.37	0.43	--	--	--	-0.32	0.32	--
**Age**	-0.30	-0.26	-0.30	-0.21	-0.41	-0.41	0.36	--	--	--
**BAI**	--	--	-0.20	--	--	--	--	0.32	--	0.30
**BDI**	--	--	-0.22	--	--	--	--	0.36	--	0.33
**MCQT**	--	--	--	--	0.25	0.23	--	--	--	--
**EPWORT**	--	--	--	--	--	--	--	0.25	--	0.25
**STIME**	--	0.25	--	0.24	0.24	0.21	--	--	--	--
**ETIME**	--	--	--	--	--	--	--	--	0.23	--
**SL**	-0.23	--	-0.24	--	--	--	--	0.31	--	0.22
**SE-SD**	--	--	--	-0.22	--	--	--	--	--	--

Notes: All correlation coefficients are statistically significant (p<0.05); Sleep time (STime); End time (ETime); Sleep latency (SL); Sleep efficiency standard deviation (SE-SD); Sleep fragmentation (SF); Number of awakenings (AW); Beck anxiety inventory (BAI); Beck depression inventory (BDI); Munich chronotype questionnaire (MCTQ); Automated working memory assessment (AWMA): mean composites of verbal short-term memory scores (V-STM), visuospatial short-term memory scores (VS-STM), verbal working memory scores (V-WM), and visuospatialworking memory scores (VS-WM); D2 test: total number of characters processed (TN), total correctly processed (TN-E), and percent of errors (E%); Psychomotor vigilance task (PVT): reaction time (RT); false start (FS).

**Table 5:** Partial correlations, controlling age, between cognitive tests, and actigraphic parameters separated by sex.

WOMEN	TST	SL	SE	WASO	SE-DP	STIME	ETIME	MIDPOINT	BAI
**TN**	--	--	--	--	--	*	*	*	--
**TN-E**	--	--	--	--	--	*	0.29	0.29	--
**E%**	--	--	--	--	--	*	-0.33	-0.29	--
**MEN**	TST	SL	SE	WASO	SE-DP	STIME	ETIME	MIDPOINT	BAI
**TN**	--	-0.47^#^	--	--	--	--	--	--	--
**TN-E**	--	-0.46^#^	--	--	--	--	--	--	--
**E%**	--	0.52^#^	--	*	--	--	0.34	--	0.31
**V-WM**	--	*	*	*	*	-0.38^#^	-0.53^#^	-0.50^#^	-0.36
**VS-WM**	--	--	--	--	-0.42^#^	--	--	--	--

Notes: All correlation coefficients are statistically significant (p<0.05); SF and AW not showed any significant correlation;

* When not controlling for age, this relationship becomes significant (p<0.05); #When controlling for anxiety symptom, this relationship also maintain statistical significance (p<0.05).

### 3.4. Cognitive tasks

The results of the sustained attention D2-test, the four composites of AWMA and PVT parameters of the women and men are shown in Table 5. There were a significant main effect of group for AWMA scores [Wilks’ Λ=0.77, F(4, 92)=6.61, p<0.01] and PVT parameters [Wilks’ Λ=0.79, F(3, 82)=6.91, p<0.01]. The post hoc difference between the groups are shown in the Table 6. The results of D2-test did not show differences between the groups [Wilks’ Λ=0.97, F(3, 90)=0.75, p=0.52] (Table 6).

**Table 6:** Scores of cognitive tasks by sex.

	WOMEN	MEN	d	*p-*value	R^2^
**AWMA** **V-STM** **VS-STM** **V-WM** **VS-WM**	**(n=50)** 26.0± 3.4 27.9± 3.1 19.0± 2.7 20.8± 4.2	**(n=47)** 26.9± 3.4 30.9± 4.0* 21.6± 3.7* 25.3± 5.3*	-0.35 -0.78 -0.61 -084	0.17^a,^ (0.14)^b^ <0.01^a,b^ <0.01^a,b^ <0.01^a,b^	16% 23% 29% 26%
**D2 TEST** **TN** **TN-E** **E%**	**(n=49)** 474.4± 68.1 343.5± 98.3 28.9± 11.5	**(n=45)** 473.8± 83.2 349.0± 117.8 28.4± 13.1	0.39 0.35 12.8	0.97^a^ (0.96)^b^ 0.80^a^ (0.79)^b^ 0.83^a^ (0.81)^b^	22% 21% 18%
**PVT TEST** **RT** **FS** **Lapses**	**(n=49)** 296.2± 56.8 0.6± 1.0 2.6± 6.3	**(n=37)** 265.0± 24.7* 3.0±5.2* 1.0± 1.0	0.69 -0.85 0.43	<0.02^a,b^ <0.01^a,b^ 0.13^a^ (0.11)^b^	23% 18% 15%

Notes:

a MANOVA;

b ANCOVA adjusted for age, BDI, TST, SL, and SF; Cohen’s d effects sizes were considered positive when W>M and negative when M>W; R^2^ considering age, BDI, TST, SL, and SF; Automated working memory assessment (AWMA): Mean composites of verbal short-term memory scores (V-STM); Visuospatial short-term memory scores (VS-STM); Verbal working memory scores (V-WM); and Visuospatial working memory scores (VS-WM); D2 test: total number of characters processed (TN); Total correctly processed (TN-E); Percent of errors (E%); Psychomotor vigilance task (PVT): reaction time (RT); false start (FS).

Given the R^2^ value, 14% of the variability of the variable V-STM is explained by the covariates (Table 6). Based on the type III sum of squares, the age (p<0.01) and SL (p<0.05) brought significant information to explain the variability of the V-STM. In relation to VS-STM, 23% of the variability is explained by the covariates age (p<0.01) and sex (p<0.001) being the last was the most influential.

The results of WM tests showed that the 29% and 26% of the variability of the dependent variables V-WM and VS-WM were explained by the explanatory variables age (p<0.01) and sex (p<0.01), age was the most influential to V-WM and sex to VS-WM.

Considering the same predictors (age, sex, LS, TST, and SF) the R^2^ value of D2-test parameters it was around 20% (see Table 6) in that the covariate age (p<0.01) the covariate that brought significant information to explain the variability of the TN, TN-E, and TE%.

Given the R^2^ value, 23%, 18%, and 15% of the variability of the dependent variables RT, FS, and lapse, respectively, were explained by the covariates. The covariate sex (p<0.05) brought significant information for RT and FS, whereas BDI score (p<0.05) it was significant for RT and lapse parameters. The covariate TST (p<0.05) brought significant information only to FS parameter.

## DISCUSSION

The main objective of this research was to evaluate the interaction between sleep quality and sex on cognitive performance. In addition, we evaluated the relation of these variables with depression and anxiety. Our results suggest that impaired sleep is associated with cognitive deficits and feelings of anxiety and depression, with men presenting more pronounced cognitive impairments.

Our findings are consistent with previous literature showing that women typically have longer and less-fragmented sleep than men. It is not clear if the biological need for sleep is different between women and men, although, sex differences have recently been reported in sleep habits and preferences^36^. Women spend more time in bed, and seem to have more complaints about poor sleep quality than men, although this perception is not reflected by objective measures of sleep pattern^37^. They sleep objectively better than men, presenting a lower percentage of stage 1, and a higher percentage of slow wave sleep^2,37^. Mong and Cusmano (2016)^38^ described sex differences in various aspects of sleep of animals and humans. They suggested that the sex differences are predominantly incurring to the effects of ovarian sex steroids in females, and that would not be different in humans.

In addition, our results revealed that women present discreet, yet significantly higher, relative amplitude and interdaily stability than men. This suggests that women perform more motor activities during the day and/or low movements during the night and have a greater regularity of wake-sleep rhythm. Furthermore, women displayed earlier bedtimes and sleep midpoints than men, despite this difference not being observed in the subjective reports. These sex-dependent disparities in bedtime have previously been described in college students^39^ and have also been shown to be independent of both marital status and whether a child is at home^40^. Cain et al. (2010)^41^ reported that women have an earlier onset of melatonin peak levels and a higher melatonin amplitude than men. This group also showed that a morning preference is associated with a shorter intrinsic circadian period and is described more in women than in men^42^.

Poor sleep quality was associated with worse performance in the visual sustained attention and memory tests, and more complaints of anxiety and depression. Anxiety results in attentional resources being allocated to irrelevant distractors thereby impairing attentional control, and consequently affecting the process of memory^43^. Visuospatial ability was more resistant to the effect of impaired sleep quality, and there was a smaller decline in V-STM. However, there were moderate degree correlation between VS-WM and irregular SE. Remarkably, in our study when the men and women were analyzed separately, the correlation becomes significant only in the men group. The visual focused attention impairment was maintained even when controlling the anxiety scores. The main complaints of poor sleepers in our study were difficulty in falling asleep or maintaining sleep during the night, still that it is not possible to evaluate the depth of sleep without polysomnography. However, despite men having less sleep than women, the main sleep parameter associated with low scores was SL. Interestingly, we also found that later bedtime and wake times were associated with low cognitive test scores in men. In fact, the sleep onset time of the men was after midnight, and the combination of circadian misalignment and chronic sleep loss may have contributed to cognitive impairment.

Although chronic insomnia has an important socioeconomic impact, the cognitive deficit mechanisms underlying sleep loss remain poorly understood. Studies with healthy volunteers submitted to different SD paradigms and fMRI records of patients with insomnia have shown hyper- or hypofunction in several structures during the performance of memory tests^44^. These differences seem to depend on the level of relative cognitive demand required to complete specific tasks. Even so, interindividual differences and the role of sex, although poorly explored, may contribute to the inconsistent findings derived from functional neuroimaging studies.

The PVT was not affected by poor sleep quality; however, there was sex effect in our study. Pejovic et al. (2013)^45^ observed a tendency for slower reaction times in volunteers who had their sleep reduced from 8 to 6 hours/night for one week. In this study, no sex effects were observed, however, slow wave sleep at baseline was associated with less subjective sleepiness during the restriction period and greater amelioration after recovery in women. This study corroborate previous result^46^ that suggest men are more affected due to significant increases in inflammatory markers after mild sleep restriction, which are not seen in women.

Distinctive cognitive performance was observed in men and women. Our results are in agreement with the literature that men perform better in visuospatial tests^15^. There is some evidence that these differences between men and women can be increased by limiting the execution time during visuospatial tasks, whereas untimed tests allow women to demonstrate their abilities better^47^. Unlike some studies, a better performance of women in verbal tasks was not observed^15,16^. There was no difference in V-STM and the mean performance of men was slightly better than women with respect to V-WM. However, the differences between sexes in verbal tasks are frequently small, do not consistently favor females and depend on the type of verbal ability involved^47^. Studies addressing the sex difference in the performance cognitive has proposed that fluctuation of hormones during menstrual cycle influence in the memory processing^37^. However, studies on menstrual cycle-dependent changes in cognition have yielded inconsistent results^48^. Nevertheless, in our study was not controlled the menstrual cycle phase.

Further differences were observed when analyzing other cognitive abilities. Sex predicted shorter reaction times, while controlling for depressive and anxiety symptoms. Although volunteers were instructed to be “as fast as possible” at the PVT, men had shorter reaction times (~ 30ms difference) than women. However, they made more mistakes, frequently pressing the button without seeing the stimuli. Blatter et al. (2006)^49^ suggested that women, despite showing slower reaction times, maintain accuracy because they avoid impulsive responses. In terms of sustained visual attention, no difference was observed between men and women, suggesting that the sex effect in the PVT is not due to differences in visual attention. In fact, PVT performance improved significantly in women after a military training program^50^ suggesting that sex differences may be due to distinctive cognitive strategies chosen by men and women^51^.

Our results suggest that sleep disturbances may impose different cognitive impacts on men and women. Considering the sex differences in sleep patterns, separate sex-specific analyses could provide additional information on the relationship between difficulties in falling asleep and cognitive impairments. Women tend to take longer to sleep, while men appear to sleep a little less than women. These findings may explain the confusion encountered by some studies in interpreting results, especially regarding initial insomnia. In addition, differences in cognitive performance between the sexes, even in the absence of sleep complaints, may add further bias in our understanding of the interaction between insufficient sleep and cognition. Therefore, sex differences in sleep quality and cognitive skills should be taken into account in future research in this field.

### Limitations

There are some limitations in our study. Clinical exams were not done. In order to control for the presence of sleep disturbances or pathological cognitive decline, several scales were applied during the interview to exclude people with any symptoms of these conditions. Moreover, most of the volunteers had an annual medical checkup and reported being in good physical condition. Another limitation is the fact that actigraphy does not perfectly measure sleep hours when compared with the gold standard of polysomnography. In addition, it does not allow the analysis of sleep architecture. Actigraphy may overestimate SL, TST, and SE, while underestimating intermittent awakenings^52^. Despite these limitations, actigraphy provides valuable information about the wake-sleep cycle because it allows longitudinal studies to be performed without interfering with the routine of the individual. Furthermore, it permits patients with insomnia to be objectively differentiated from normal sleepers^53^.

## CONCLUSION

The sex-related differences observed in this study highlight the need to separate women and men in studies that evaluate the effect of sleep deprivation or sleep disorder on cognitive function. This is particularly important due to sex differences in sleep pattern, cognitive test performance, and reactions to external stressors.

## Abbreviations list:

Alcohol, smoking and substance involvement screening test (ASSIST)

Analysis of covariance (ANCOVA)

Automated working memory assessment (AWMA)

Awakenings (AW)

Beck anxiety inventory (BAI)

Beck depression inventory - 2^nd^ edition (BDI-II)

Body mass indices (BMI)

End time (ETime)

False start (FS)

Interdaily stability (IS)

Intradaily variability (IV)

Maximum (Max)

Mean (m)

Midpoint of sleep time (MTime)

Minimum (Min)

Multivariate analysis of variance (MANOVA)

Munich chronotype questionnaire (MCTQ)

Number of volunteers (N)

Percent of errors (E%)

Pittsburgh sleep quality index (PSQI)

Psychomotor vigilance tests (PVT)

Reaction time (RT)

Relative amplitude (RA)

Sleep deprivation (SD)

Sleep efficiency (SE)

Sleep efficiency standard deviation (SE-SD)

Sleep fragmentation (SF)

Sleep latency (SL)

Sleep time (STime)

Standard deviation (SD)

Total correctly processed (TN-E)

Total number (TN)

Total sleep time (TST)

Verbal short-term memory (V-STM)

Verbal working memory (V-WM)

Visuospatial short-term memory (VS-STM)

Visuospatial working memory (VS-WM)

Wake after sleep onset (WASO)

Wilks lambda (Wilks’ Λ)

Working memory (WM)

Years (y)
